# Historic analysis of habitat suitability for the commercially promising berry crop *Kadsura longipedunculata* in China under climate change

**DOI:** 10.1371/journal.pone.0333824

**Published:** 2025-10-23

**Authors:** Kai Chen, Zhaoqi Xie, Binsheng Luo, Jiaxing Yang, Mingli Hu, Chunsong Cheng

**Affiliations:** 1 Jiangxi Key Laboratory for Sustainable Utilization of Chinese Materia Medica Resources, Lushan Botanical Garden, Chinese Academy of Sciences, Jiujiang, China; 2 Lushan Xinglin Institute for Medicinal Plants, Jiujiang Xinglin Key Laboratory for Traditional Chinese Medicines, Jiujiang, China; 3 School of Pharmacy, Xianning Medical College, Hubei University of Science and Technology, Xianning, Hubei, China; Sun Yat-Sen University School of Geography and Planning, CHINA

## Abstract

Climate change is threatening global plant diversity, necessitating the identification of resilient species for sustainable utilization. This study presents the first comprehensive prediction integrating paleo, current, and future climate scenarios with soil and terrain variables to assess *Kadsura longipedunculata*, a cold-tolerant evergreen liana with economic and medicinal values. Using 158 validated species occurrence records and 15 key environmental variables (climate, soil, and terrain), we employed the MaxEnt model integrated with ArcGIS to predict distribution shifts across the Last Glacial Maximum (LGM; ~ 22 kyr BP), Mid-Holocene (MH; ~ 6 kyr BP), current (1970–2000), and future periods (2041–2060, 2081–2100) under SSP126 and SSP585 scenarios. Our results revealed that precipitation during the driest month (bio14) was the most critical factor influencing habitat suitability, contributing 75.9% to the model. Under current conditions, highly suitable habitats were concentrated in southeastern China (25°N–30°N), particularly in Jiangxi, Fujian, and Hunan provinces. Future projections indicated significant restructuring: firstly total suitable area showed limited change under most scenarios (<±10%), but low-suitability habitats were showed contracting substantially (>20%) under SSP126-2050s and SSP585-2090s, while medium-suitability areas were showed expanding (up to +17.0%). High-suitability habitats were showed remaining stable, and a northward migration trend of distribution centroids, and highlights both the species’ resilience in core montane habitats and its vulnerability to precipitation changes. The findings offer a scientific basis for conserving and domesticating this species, with Jiangxi Province identified as a key region for future cultivation efforts.

## 1. Introduction

More than 17,000 tree species are threatened by rapid global environmental changes [1], with climate change projected to substantially alter the distribution and extent of plant habitats [2] Studies indicate that a 2°C increase in global temperatures could reduce plant distribution ranges by 16% [3]. Such shifts are particularly detrimental to species characterized by narrow ecological niches. *K. longipedunculata*, an evergreen liana uniquely adapted to the subtropical-temperate transition zone of southeastern China [4], exemplifies this vulnerability. As a cold-tolerant species within its genus, it faces compounded pressures from concurrent climate warming and precipitation fluctuations. Consequently, it is critical to account for climate change and to identify key climatic variables when establishing new crop research and industrialization centers [5].

The genus *Kadsura* (Kaempf. ex Juss.) is native to subtropical and tropical Asia, with a distribution extending from southern China to temperate East Asia, with southern China serving as its center of diversity [6,7]. Most *Kadsura* species possess significant economic and medicinal value [8], among which *K. longipedunculata*—an evergreen climbing shrub—stands out as the most cold-tolerant. This species is predominantly distributed in subtropical monsoon humid hilly regions north of the Tropic of Cancer, including Jiangxi, Fujian, Hunan, and Guangxi provinces. Its ripe fruit is a red apocarpous berry, traditionally used to treat rheumatic arthritis, traumatic injuries, dysmenorrhea, abdominal pain, irregular menstruation, canker sores, and gastrointestinal inflammation [9,10]. The fruit is not only sweet and palatable but also rich in vitamin C, vitamin E, and essential trace elements. In rural areas, particularly in Jiangxi’s Lushan region, it is consumed as a natural wild fruit and processed into commercial products such as preserves, jams, canned foods, and syrups [11]. Currently, the market for small berry products is dominated by *Schisandra chinensis*. However, its strong sour taste limits its consumer appeal in Northeast Asia [12]. In contrast, the fruit of *K. longipedunculata* offers a refreshingly sweet flavor profile. The total acid content in its pulp and peel is merely 0.03% and 0.02%, respectively (calculated as citric acid) [13,14], significantly lower than the 4.93% total acid content found in *S. chinensis*. This confers a distinct advantage in terms of palatability. In 2021, our research team proposed the development of *Kadsura* species—particularly *K. longipedunculata*—as a promising commercial wild fruit crop, aiming to advance the healthy berry industry in the 21st century [15].

We employed the Maximum Entropy (MaxEnt) algorithm [16] to construct Species Distribution Models (SDMs), owing to its robust performance with presence-only data and limited sample sizes, a common challenge for understudied species such as *K.longipedunculata*. Unlike regression models (e.g., GLM, GAM) requiring potentially biased pseudo-absence data [17], or machine learning methods (e.g., Random Forests) prone to overfitting in small datasets [18]; MaxEnt reliably estimates species-environment relationships without these constraints. The model also outperforms simpler methods like Bioclim and ENFA by effectively capturing complex environmental interactions [19]. Its interpretable outputs—particularly variable contribution metrics and response curves—provide tangible evidence for identifying key climatic drivers and formulating conservation strategies [20].

Despite significant economic and medical importances of *K. longipedunculata*, critical knowledge gaps remain in predicting its climate-driven habitat shifts. Research has primarily prioritized investigating the species’ pharmacological properties [21–23], with considerably less attention directed toward its ecological adaptability and distribution dynamics. Furthermore, although the climate-distribution relationships of closely related species (e.g., *Schisandra chinensis*) have been investigated [24], comprehensive projections specifically for *K. longipedunculata* remain lacking. The domestication and improvement of the *Kadsura* genus necessitates the selection of a core species with broad distribution, strong stress resistance, and superior propagation traits. This study aims to fill this gap by employing the MaxEnt model, integrated with Geographic Information System (ArcGIS) technology, to evaluate the potential habitat suitability of *K. longipedunculata* under past (Last Glacial Maximum [LGM], Mid-Holocene [MH]), present (1970–2000), and future (2041–2060, 2081–2100) climate scenarios. By analyzing climatic, edaphic, and topographic variables, we identify the key environmental factors influencing the species’ geographic distribution.

To our knowledge, this is the first study to predict the effects of climate change on the suitability of *K. longipedunculata*, a cornerstone species of the *Kadsura* genus. Our overarching objective is to project its potential distribution range under current and future climate conditions, elucidating the critical environmental drivers behind shifts in its spatial distribution. These findings will provide a scientific foundation for the conservation, sustainable management, and strategic selection of optimal cultivation sites for *K. longipedunculata*.

## 2. Materials and methods

### 2.1. Acquisition and screening of species distribution information

**Fig 1:**
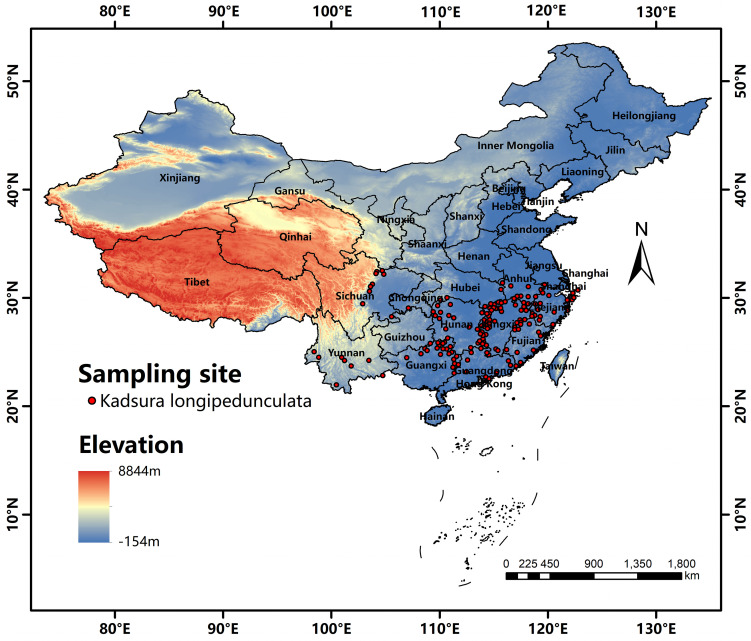
Sampling distribution area of *K. longipedunculata* within China (based on altitude map). The base map was created using open-access data from Natural Earth (https://www.naturalearthdata.com/). The depiction of China’s territory in this figure is consistent with the official standard map of the People’s Republic of China (Map Approval No.: GS(2024)0650 cited from https://cloudcenter.tianditu.gov.cn/administrativeDivision).

The occurrence records of K. longipedunculata were obtained from the Digital Herbarium of China (http://www.cvh.ac.cn) and the National Specimen Information Infrastructure of China (http://www.nsii.org.cn). From an initial collection of 1,177 records across mainland China. Records were excluded based on the following criteria: collection dates prior to 1980, unlabeled geographic locations, poor image quality, or spatial duplication. For records with detailed geographical descriptions but lacking specific coordinates, precise locations were determined using the Baidu Map coordinate picker (https://api.map.baidu.com/lbsapi/getpoint/index.html), resulting in a validated subset of 263 occurrence records. To minimize spatial autocorrelation and model overfitting, spatial thinning was performed using ArcGIS 10.4. A 1 km buffer was applied around each occurrence point, with only one randomly selected point retained within each buffer zone. Following this thinning procedure, and subsequent validation through field surveys, satellite imagery verification, and literature records [25–28], a final set of 158 validated occurrence records was obtained. The distribution of records covered the following provinces: Jiangsu (1), Anhui (9), Zhejiang (19), Jiangxi (46), Fujian (11), Hubei (2), Hunan (20), Guangdong (9), Guangxi (23), Sichuan (9), Chongqing (1), and Yunnan (8) (Fig 1).

### 2.2. Acquisition and screening of environmental factors

Nineteen bioclimatic variables and a foundational Digital Elevation Model (DEM) dataset were obtained from the WorldClim database (version 2.1; 30-arcsecond resolution; http://www.worldclim.org) [29]. The remaining terrain factors (slope, aspect) were subsequently derived from this DEM using the Surface Analysis tools within ArcGIS 10.4. To prevent overfitting and ensure model parsimony, variables exhibiting correlation coefficients exceeding 0.8 were excluded during preliminary screening, retaining them with higher contributions to the model. The nineteen climatic factors in the last glacial maximum (LGM), the middle Holocene (MH), and the present (1970–2000), the future 2050s (2041–2060) and the future 2090s (2081–2100) were applied to the analysis of this study. Future climate projections were derived from the BCC-CSM2-MR (Beijing Climate Center Climate System Model) GCM of the Sixth International Coupled Model Comparison Project (CMIP6) under the Shared Socioeconomic Pathways (SSPs). This model was selected for its reliable performance in simulating East Asian climate patterns. SSP126 (low emissions scenario) and SSP585 (high emissions scenario) were selected to represent the most optimistic and pessimistic future greenhouse gas emission scenarios [30]. Additionally, eleven soil variables were acquired from the Harmonized World Soil Database (version 1.2; 30-arcsecond resolution; http://www.fao.org/soils-portal/data-hub/en/) [31]. This resulted in a total of 33 independent environmental variables for subsequent analysis (Table 1).

**Table 1:** Description of environmental variables.

Variable	Description	Unit	Variable	Description	Unit
bio_1	Annual average temperature	°C	bio_18	Precipitation in the warmest quarter	mm
bio_2	Monthly mean temperature	°C	bio_19	Coldest seasonal precipitation	mm
bio_3	Isothermality	—	awc_class	Soil available water content	—
bio_4	Temperature Seasonality (standard deviation ×100)	—	s_caco3	Carbonate or lime content of subsoil	%
bio_5	Maximum temperature in warmest month	°C	s_clay	Clay content in the subsoil	%
bio_6	Minimum temperature in coldest month	°C	s_oc	Subsoil organic carbon content	%
bio_7	Annual temperature range	°C	s_ph_h2o	Subsoil pH	—
bio_8	Average wettest season temperature	°C	s_sand	Sediment content in the subsoil	%
bio_9	Driest quarterly mean temperature	°C	t_caco3	Topsoil carbonate or lime content	%
bio_10	Average temperature of the warmest quarter	°C	t_clay	Clay content in the upper soil	%
bio_11	Average temperature of coldest quarter	°C	t_oc	Topsoil organic carbon content	%
bio_12	Average annual precipitation	mm	t_ph_h2o	Topsoil pH	—
bio_13	Wettest monthly precipitation	mm	t_sand	Upper soil sand content	%
bio_14	The driest monthly precipitation	mm	aspect	Aspect	**°**
bio_15	Seasonal variation of precipitation	—	elev	Elevation	m
bio_16	Wettest quarterly precipitation	mm	slope	Slope	**°**
bio_17	Driest quarterly precipitation	mm	—	—	—

Unit “—” indicates that no unit is required.

**Fig 2:**
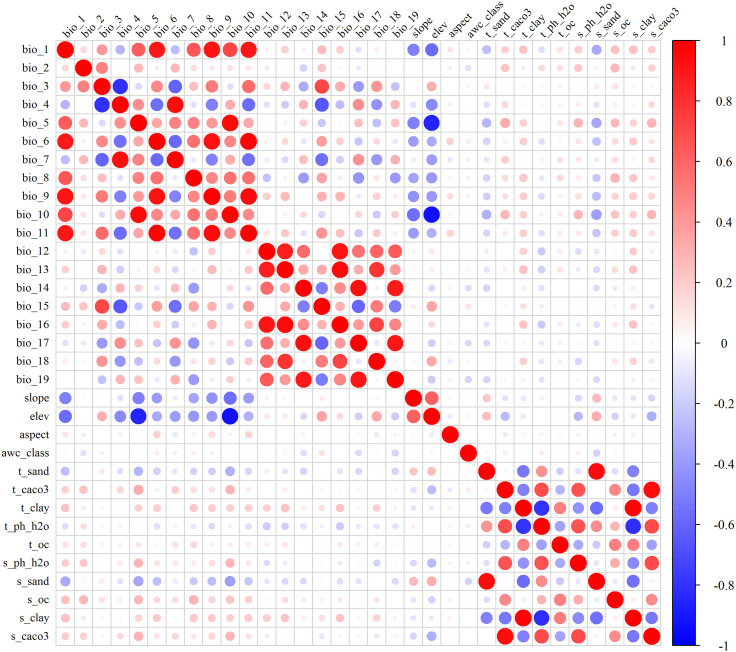
Correlation Heatmap of Environmental Covariates (The red circles are for positive correlations and the blue ones for negative correlations. The stronger the correlation, the larger the size of the circle and the darker the color.).

Highly correlated environmental variables can lead to model overfitting [20]. To ensure predictive accuracy, Spearman correlation analysis was performed on all environmental variables using IBM SPSS Statistics 21. The Jackknife method was then employed to preliminarily assess variable contributions. From pairs exhibiting correlation coefficients exceeding 0.8, the variable with the highest contribution was retained while others were discarded (Fig 2) [32,33]. This process yielded a final set of 15 variables for constructing the *K. longipedunculata* distribution model. These comprised 10 climatic factors (bio_2, bio_3, bio_4, bio_6, bio_8, bio_10, bio_14, bio_15, bio_16, bio_18), 5 soil factors (t_clay, t_caco3, t_sand, t_oc, s_oc), and 2 terrain factors (aspect, elev).

### 2.3. Construction of the MaxEnt model

The acquired sampling point data of *K. longipedunculata* and the effective environmental variables were imported into the MaxEnt software (Version 3.4.3) to predict the potential suitable habitat distribution of *K. longipedunculata*. The modeling parameters were configured as follows: the bootstrap method was employed for sampling, the output format was set to Logistic, and 75% of the distribution points were randomly selected as the training set, while the remaining 25% were allocated as the test set. For each training partition, the model underwent 100 iterations and was repeated 10 times [34,35]. Model predictive accuracy was assessed using the Area Under the Receiver Operating Characteristic Curve (AUC). Ranging from 0 to 1, AUC serves as a key performance metric—values approaching 1 indicate higher predictive accuracy, with scores exceeding 0.9 signifying excellent model performance [36]. Habitat suitability for *K. longipedunculata* was classified using the Maximum Training Sensitivity plus Specificity (MTSPS) logistic threshold. This method optimally balances sensitivity and specificity, avoiding subjective threshold selection and aligning with best practices in species distribution modeling [20]. Habitats were categorized into four suitability levels: Unsuitable (0–MTSPS), Low Suitability (MTSPS–0.3), Medium Suitability (0.3–0.5), and High Suitability (0.5–1), with the area of each level quantified. Subsequently, the centroid coordinates of potential suitable habitat distribution maps for each period were calculated, and the displacement distance and bearing were quantified using the Spatial Statistics tools in ArcGIS 10.4.

## 3. Results and analysis

### 3.1. Model accuracy analysis

**Fig 3:**
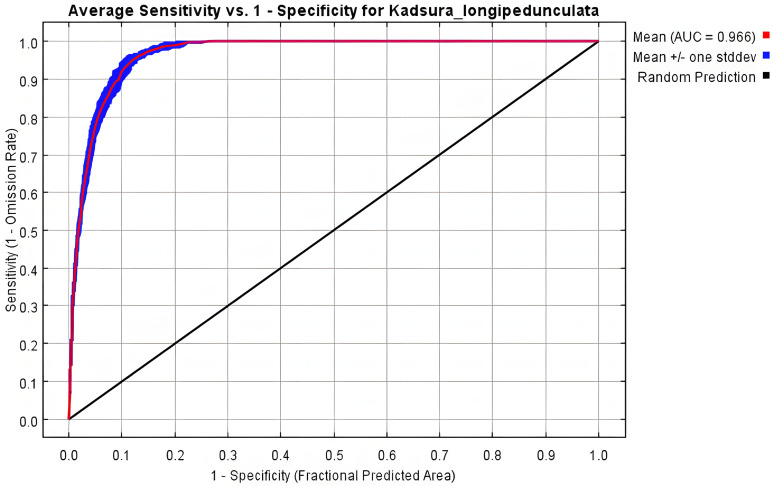
ROC curve (Mean AUC = 0.966) of *K. longipedunculata*’s MaxEnt adaptive zone model.

The potential distribution range of *K. longipedunculata* was simulated and predicted using the MaxEnt software, with the average Receiver Operating Characteristic (ROC) curve calculated from 10 independent iterations. As shown in Fig 3, the mean training Area Under the Curve (AUC) value was 0.966, exceeding the threshold of 0.9, indicating high model accuracy. This confirms the model’s robustness for identifying potential suitable habitats for *K. longipedunculata*.

### 3.2. Environmental factor analysis

To determine the influence of individual environmental variables on model construction, the Percent Contribution and Permutation Importance of the 15 selected variables were analyzed within the MaxEnt framework. As presented in Table 2, precipitation of the driest month (bio_14) exhibited the highest contribution (75.9%), identifying it as the predominant influencing factor. Although precipitation of the wettest quarter (bio_16), elevation (elev), and minimum temperature of the coldest month (bio_6) demonstrated more modest contributions (all < 5%), their considerable permutation importance values indicate they nonetheless constitute significant factors influencing the distribution of *K. longipedunculata*. (Table 2).

**Table 2:** Percent contribution and permutation importance of environmental variables in the MaxEnt model for *K. longipedunculata.*

Variable	Description	Units	Percent contribution	Permutation importance
bio_14	The driest monthly precipitation	mm	75.9	19.6
bio_16	Wettest quarterly precipitation	mm	4.1	25.8
elve	Elevation	m	4.1	7.6
bio_6	Minimum temperature in coldest month	°C	2.9	16.3
bio_8	Average wettest season temperature	°C	1.8	0.7
aspect	Aspect		1.7	3.3
bio_4	Temperature Seasonality (standard deviation ×100)	—	1.2	2.3
bio_3	Isothermality	—	1.1	2.3
t_caco3	Topsoil carbonate or lime	%	1.1	2.3
t_clay	Clay content in the upper soil	%	1.1	2.3
bio_15	Seasonal variation of precipitation	—	1.1	3.3
bio_18	Precipitation in the warmest quarter	mm	0.9	4.3
bio_2	Monthly mean temperature	°C	0.9	3.4
bio_10	Average temperature of the warmest quarter	°C	0.5	3.7
s_oc	Subsoil organic carbon content	%	0.5	1.2
t_sand	Upper soil sand content	%	0.5	1.5
t_oc	Topsoil organic carbon content	%	0.3	0.7

**Fig 4:**
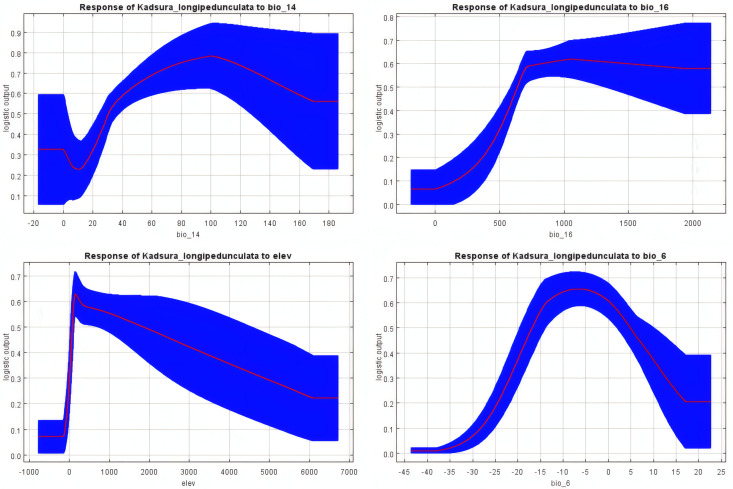
Response curves of key environmental variables (bio_14, bio_16, elev, bio_6) influencing the distribution probability of *K. longipedunculata.*

Based on the response curves of environmental variables to distribution probability generated by the MaxEnt model (Fig 4), the suitable ranges of key environmental variables for *K. longipedunculata* potential distribution were determined. The optimal ranges (suitability >0.5) for bio_14, bio_16, elev, and bio_6 are 40–180 mm, 500–2000 mm, 200–2000 m, and −15–5 °C, respectively.

Crucially, the synergistic effect of bio_14 and bio_16 reveals the species’ dichotomous water requirements: avoidance of dry-season stress coupled with an adequate moisture supply during the growth season. Elevation (elev) indirectly mitigates dry-season water limitations by modulating local climate (e.g., orographic precipitation in cloud belts), with its optimal range (200–2000 m) aligning with humid montane regions. Despite defining the lower thermal tolerance threshold (−15–5 °C), the minimum temperature of the coldest month (bio_6) exhibited a low contribution rate (2.9%), confirming that moisture constraints predominantly govern distribution within the core range. In summary, the distribution of *K. longipedunculata* is primarily driven by water balance (centered on dry-season security), secondarily modulated by topographic elevation, and further limited by low temperatures.

### 3.3. Current distribution prediction

**Fig 5:**
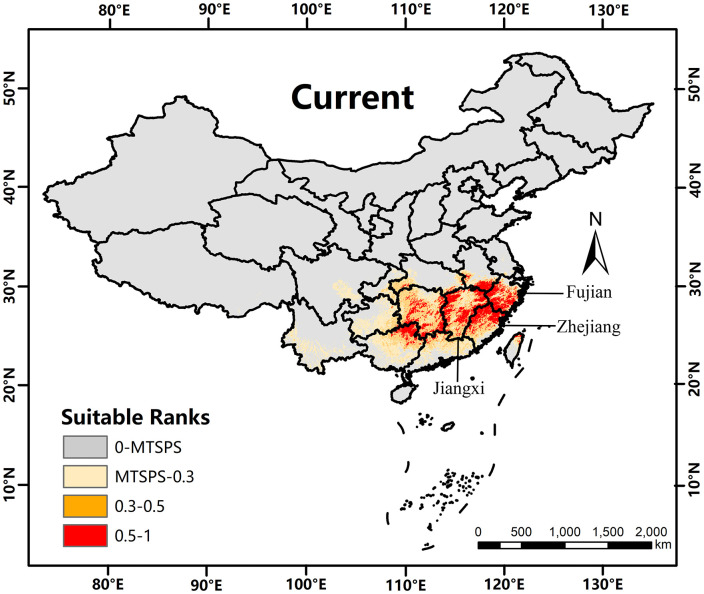
Distribution of Suitable Habitats for *K. longipedunculata* under Current Conditions. The base map was created using open-access data from Natural Earth (https://www.naturalearthdata.com). The depiction of China’s territory in this figure is consistent with the official standard map of the People’s Republic of China (Map Approval No.: GS(2024)0650 cited from https://cloudcenter.tianditu.gov.cn/administrativeDivision).

The simulated distribution pattern of *K. longipedunculata* under current climatic conditions is illustrated in Fig 5. Non-suitable habitats are shaded gray, while low-, medium-, and high-suitability habitats are represented in yellow, orange, and red, respectively. The model shows that the primary distribution range of *K. longipedunculata* lies between 25°N and 30°N, with a concentration of suitable habitats in southeastern China. This distribution aligns closely with the species’ natural range as documented in the *Flora of China*. The high-suitability areas are predominantly distributed across the provinces of Anhui, Jiangxi, Fujian, Zhejiang, Hunan, and Guangxi provinces, with extensive coverage along the mountainous regions near provincial boundaries. The medium-suitability areas surround the high-suitability zones, while the low-suitability areas are distributed around both the high- and moderate-suitability regions.

### 3.4. Past and future distribution predictions

**Fig 6:**
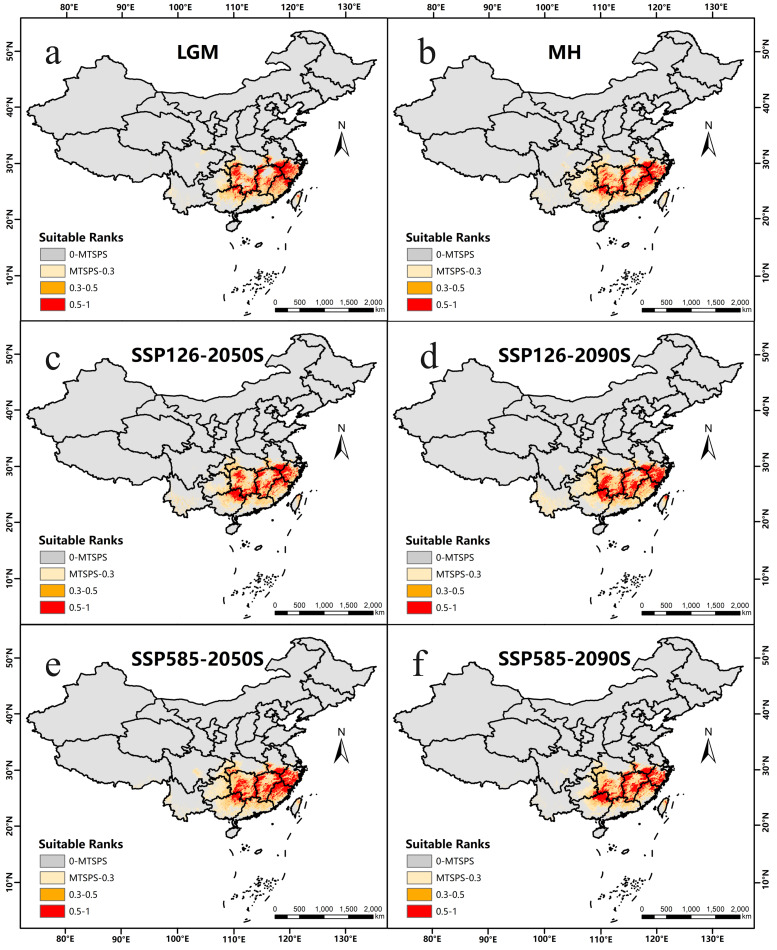
Distribution of suitable habitats for *K. longipedunculata* under past (LGM, MH), current, and future (SSP126-2050s, SSP126-2090s, SSP585-2050s, SSP585-2090s) climate scenarios. The base map was created using open-access data from Natural Earth (https://www.naturalearthdata.com). The depiction of China’s territory in this figure is consistent with the official standard map of the People’s Republic of China (Map Approval No.: GS(2024)0650 cited from https://cloudcenter.tianditu.gov.cn/administrativeDivision). (a: Distribution of Suitable Habitats for *K. longipedunculata* under LGM Conditions; b: Distribution of Suitable Habitats for *K. longipedunculata* under MH Conditions; c: Distribution of Suitable Habitats for *K. longipedunculata* under SSP126-2050S Conditions; d: Distribution of Suitable Habitats for *K. longipedunculata* under SSP126-2090S Conditions; e: Distribution of Suitable Habitats for *K. longipedunculata* under SSP585-2050S Conditions; f: Distribution of Suitable Habitats for *K. longipedunculata* under SSP585-2090S Conditions).

This study projected the potential distribution of *K. longipedunculata* across six distinct time periods in China. MaxEnt simulations revealed the dynamic shifts in suitable habitat distribution from the Last Glacial Maximum (LGM) to the present and under future climate scenarios (Fig 6). Compared to the LGM, the total suitable habitat area during the Mid-Holocene (MH) exhibited a slight reduction (approximately 10%). However, this change was primarily driven by a nearly 50% contraction in Low Suitability habitat, while areas of medium and high Suitability remained relatively stable (Figs 6a-b, Table 3). This pattern suggested that post-glacial warming predominantly led to the contraction of peripheral (Low Suitability) distribution zones, while the core distribution area (medium/high Suitability) demonstrated resilience.

**Table 3:** Temporal Variations in Suitable Habitat Area and Its Percentage Change Relative to Present for *K. longipedunculata.*

Period		Low suitability habitat		Medium suitability habitat		High suitability habitat		Total suitability habitats	
		Area (×10³ km²)	Δ% (vs Current)	Area (×10³ km²)	Δ% (vs Current)	Area (×10³ km²)	Δ% (vs Current)	Area (×10³ km²)	Δ% (vs Current)
LGM		504.01	19.94%	403.26	8.20%	240.99	−13.99%	1148.26	7.01%
MH		261.13	−37.86%	394.41	5.83%	254.32	−9.23%	909.86	−15.21%
Current		420.23	0.00%	372.69	0.00%	280.17	0.00%	1073.09	0.00%
2050S	SSP126	305.33	−27.34%	430.07	15.40%	261.48	−6.67%	996.88	−7.10%
	SSP585	421.04	0.19%	436.18	17.04%	301.53	7.62%	1158.75	7.98%
2090S	SSP126	438.49	4.35%	406.04	8.95%	283.58	1.21%	1128.11	5.13%
	SSP585	322.74	−23.20%	373.39	0.19%	279.58	−0.21%	975.71	−9.07%

Compared to the current climate baseline (1970–2000), model projections indicate that the overall magnitude of change in the total suitable habitat area for *K. longipedunculata* remains limited across future periods (absolute change <10% under most scenarios), suggesting relative stability in its distribution extent under future climates (Fig 6c-f, Table 3). However, significant restructuring of its internal suitability hierarchy (proportions of Low, Medium, and High Suitability areas) and spatial configuration occurred, with distinct adjustment patterns emerging across different emission scenarios and time periods.

Under the low emission scenario (SSP126) in the 2050s, The total suitable area decreased slightly (−7.1%). This was driven primarily by a substantial contraction in low suitability habitat (−27.3%) and a significant reduction in high suitability area (−6.7%), while medium suitability area expanded markedly (+15.4%) (Fig 6c, Table 3). This suggests that under moderate warming, portions of the current high suitability zone may transition to medium suitability, while some low suitability areas or newly suitable regions shift into the medium suitability class. Projecting to the 2090s under the same low emission pathway, Total suitable area increased modestly (+5.1%). Expansions occurred across all suitability levels, with medium suitability showing the most pronounced relative growth (+8.9%) (Fig 6d, Table 3). This indicates that under a low-emission pathway in the long term, climatic conditions may exert a marginally favorable influence on distribution. In contrast, the high emission scenario (SSP585) paints a different picture for the 2050s. Total suitable area increased significantly (+8.0%), characterized by synchronous expansion across all suitability levels. Growth was particularly notable in medium suitability (+17.0%) and high suitability (+7.6%) zones (Fig 6e, Table 3). This demonstrates that during the initial phase of strong greenhouse forcing, climate warming may transiently expand the species’ suitable habitat range. However, by the 2090s under this extreme emission trajectory, Total suitable area contracted substantially (−9.1%). This decline was primarily driven by a sharp reduction in low suitability habitat (−23.2%), accompanied by a marginal decrease in high suitability area (−0.2%), while medium suitability area showed negligible change (+0.2%) (Fig 6f, Table 3). Under this scenario, by the end of the century, extreme climate change is projected to cause significant habitat contraction, with peripheral habitats (low suitability) facing substantial contraction risks.

Integrated analysis reveals that Low Suitability habitats exhibit substantial contractions under SSP126-2050s and SSP585-2090s, highlighting the vulnerability of peripheral distribution zones. Conversely, Medium Suitability areas demonstrate an expansion trend across all future scenarios except SSP585-2090s, suggesting their potential pivotal role in sustaining the species’ future distribution. High Suitability habitats remain largely stable, with the mountainous borders of Jiangxi and adjacent provinces consistently identified as the core high-suitability area in both current and projected distributions (Figs 5, 6), indicating strong climatic resilience.

### 3.5. Changes in the distribution center of *K. longipedunculata*

**Fig 7:**
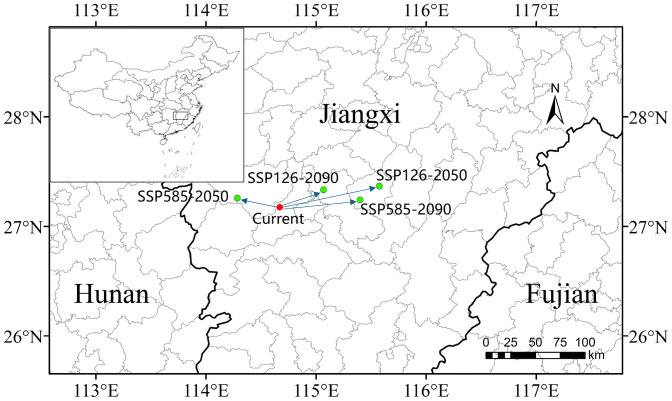
Shifts in the distribution centroid of *K. longipedunculata* under different climate scenarios. The base map was created using open-access data from Natural Earth (https://www.naturalearthdata.com). The depiction of China’s territory in this figure is consistent with the official standard map of the People’s Republic of China (Map Approval No.: GS(2024)0650 cited from https://cloudcenter.tianditu.gov.cn/administrativeDivision).

Model projections indicate that the distribution centroid of *K. longipedunculata* remains stable within central Jiangxi Province (27°–28°N, 114°–116°E) under current and future climate scenarios yet exhibits county-scale shifts. Under SSP585-2050s, the centroid migrated northward approximately 50 km to Anfu County, consistent with the general pattern of climate-warming-driven poleward migration. However, in SSP585-2090s, the centroid reversed course to Yongfeng County, a phenomenon directly linked to the concurrent drastic contraction of Low Suitability habitat (−23.2%) and intensified drought stress (reduced bio_14), demonstrating that moisture limitation can substantially counteract temperature-driven northward migration potential. Notably, across all scenarios, the centroid remains anchored in the mountainous regions of central Jiangxi (specifically Yongfeng, Anfu, and Jishui counties), confirming the long-term stability of the core high-suitability zone. This stability is likely maintained through topographic buffering, where elevation-mediated regulation of local hydrothermal conditions mitigates the impacts of climate change. This migration trajectory underscores the critical conservation priority of central Jiangxi while signaling elevated risks from peripheral habitats (Fig 7).

## 4. Discussion

### 4.1. Influence of environmental factors on the distribution of *K. longipedunculata*

Ecologically, identifying the environmental variables that govern the spatial distribution of species is fundamental for understanding their habitat preferences and conservation needs [37–39]. This study is the first to quantitatively demonstrate, via species distribution modeling, that precipitation of the driest month (bio_14) is the paramount limiting factor determining the geographic distribution pattern of *K. longipedunculata*, contributing dominantly (75.9%) and significantly exceeding the contributions of temperature and other variables. This finding stems from the species’ inherent physiological vulnerability: as an evergreen liana with shallow roots and large leaves, *K. longipedunculata* exhibits high sensitivity to drought stress [40–42]. The plant’s survival and reproduction are critically dependent on winter precipitation, a time when it remains dormant. Adequate soil moisture during this period is essential not only for maintaining dormancy vigor but also serves as the key environmental cue for breaking seed dormancy. Studies on closely related species have shown that winter moisture plays a dual role: facilitating the morphological differentiation of immature embryos (increasing embryo length proportion from 30% to 95%) and regulating critical metabolic shifts (e.g., soluble substrate depletion, GA/ABA homeostasis). Under artificial drought stress, seed germination was inhibited by 46%, while the alleviating effect of exogenous GABA(γ-aminobutyric acid) further emphasizes the critical role of water [43–45]. Empirical observations in Qingyuan County further corroborate that the spatiotemporal distribution of winter precipitation directly dictates spring germination success rates [46].

In addition to climatic factors, non-climatic topographic elements emerge as secondary drivers that mitigate water stress by regulating local hydrothermal conditions. Elevation (elev), a key topographic factor, exhibits an optimal range (200–2000 m) that aligns precisely with orographic precipitation belts. At higher elevations, enhanced orographic precipitation (e.g., cloud/fog condensation) and reduced evaporation rates help alleviate dry-season moisture stress (bio_14) [47].

This cascading “moisture-dominance with topographic modulation” mechanism collectively explains core distribution stability: complementary precipitation factors (e.g., bio_16 ensuring growth-season moisture) synergistically maintain water balance with terrain features. This finding is consistent with studies on related taxa like *Schisandra chinensis* confirming precipitation as the primary distribution constraint [48,49].

### 4.2. Spatial-geographical analysis of suitable habitats

Multi-temporal model simulations reveal that while the Last Glacial Maximum (LGM) exhibited a larger total suitable habitat area than the Mid-Holocene (MH), it contained a substantially higher proportion of low suitability habitat (43.9% vs. 28.7%), predominantly distributed across arid plains. Paleoecological evidence suggests these areas likely represented non-sustainable habitats—supporting ephemeral germination events but failing to establish viable populations due to subsequent moisture stress [50]. Conversely, during the MH period, despite increased precipitation, enhanced evapotranspiration driven by rising temperatures reduced water availability in low-elevation marginal zones, forcing a contraction of suitable habitats toward mountainous regions [51].

Projecting into the future, climate change induces complex patterns of simultaneous expansion and contraction in suitable habitats. Low suitability areas undergo substantial reductions (>20%) under SSP126-2050s and SSP585-2090s scenarios, while showing marginal increases (<5%) in SSP126-2090s and SSP585-2050s. Conversely, medium suitability habitats exhibit consistent expansion across all four scenarios (+0.19% to +17.04%). High suitability areas display minor fluctuations (<±8%). This pattern likely reflects the integrated effects of climatic variables: fluctuations in low suitability zones are governed by precipitation (bio_14, bio_16) and temperature (bio_6). Increases under SSP126-2090s and SSP585-2050s potentially arise from improved precipitation parameters offsetting warming-induced aridity in some regions, or from warming-induced suitability gains in colder zones masking underlying moisture risks. Conversely, under SSP126-2050s and SSP585-2090s, climate change exceeds tolerance thresholds, triggering significant low suitability habitat decline [52]. The widespread expansion of medium suitability areas indicates that climate warming facilitates the transition of low suitability zones into medium suitability status, establishing them as critical zones for distributional reorganization. The remarkable stability of mountainous high suitability habitats (fluctuation <8%) demonstrates the universal buffering capacity of topography against climatic variability [53].

The spatial projections of this study reveal significant geographic expansion of potential suitable habitat for *K. longipedunculata* under specific future climate scenarios. Comparative analysis of Figs 5 and 6 indicates that substantial new suitable areas are projected to emerge in western and southern Hunan Province, northern Guangxi Province, and particularly along their border regions specifically within the Xuefeng Mountains, Bashili Dananshan, Yuecheng Ling, and Haiyang Shan ranges. These potential new suitable habitats are critically important: they represent probable natural migration pathways or potential new cultivation areas for the species under future climate change, providing essential spatial references for formulating conservation strategies and guiding industrial development.

### 4.3. Potential changes in altitudinal distribution range

Model projections reveal marked altitudinal shifts in the distribution of *K. longipedunculata* under climate change, primarily mediated through warming impacts on key limiting factors, such as precipitation (bio_14) and temperature (bio_6). Under low-emission scenarios (SSP126), relatively moderate warming may facilitate moderate upward expansion to higher elevations, while moisture stress at low-elevation margins remains partially tolerable or offset. During the initial phase of high emissions (SSP585-2050s), intense warming is projected to concurrently deteriorate precipitation regimes at low elevations and rapidly enhance suitability at higher elevations, driving significant upward migration and expansion of medium and high suitability habitats into montane zones. By the late high-emission period (SSP585-2090s), extreme warming and potential aridification will trigger extensive loss of low-elevation marginal habitats, forcing further range contraction toward higher elevations. However, this upward shift is constrained by diminishing mountain area, impoverished soil, and potential moisture limitations at high elevations, ultimately failing to compensate for low-elevation losses. This cascading effect will lead to a net contraction in total suitable habitat and elevate risks of habitat fragmentation [54].

### 4.4. Conservation and domestication of *K. longipedunculata*

Integrating projected shifts in the spatial distribution of suitable habitats, altitudinal migration trends across climate scenarios, and centroid displacement characteristics, we propose comprehensive conservation and adaptive management strategies. These prioritize: (1) enhanced in situ conservation within core areas of central Jiangxi (specifically Yongfeng, Anfu, and Jishui counties anchoring the centroid); (2) designating newly identified suitable habitats in western Hunan and northern Guangxi mountainous regions as candidate sites for assisted migration or novel cultivation; (3) monitoring population dynamics across altitudinal gradients and preserving elevational habitat corridors within core zones; and (4) prioritized germplasm collection from northern marginal populations and key counties implicated in centroid shifts (e.g., Yongfeng, Anfu) for breeding drought-tolerant varieties. All measures should be dynamically adjusted based on scenario-specific projections under divergent emission pathways (SSP126/SSP585) to implement tailored interventions that enhance the climate resilience of *K. longipedunculata*.

### 4.5. Limitations of the study

While climate, soil, and topographic factors employed in this study effectively capture the primary environmental requirements of *K. longipedunculata* and enable the prediction of its potential distribution, these variables fail to incorporate critical ecological and anthropogenic factors that directly influence its survival and dispersal. As a forest-dependent liana, the distribution and population viability of *K. longipedunculata* are fundamentally determined by specific habitat structural attributes, including canopy openness, proximity to forest edges, and intensity of anthropogenic disturbances [55–57]. Previous research has shown that neglecting these variables can result in a substantial overestimation of suitable habitat area, potentially exceeding 50% [58,59]. The current distribution range of K. longipedunculata predominantly encompasses sparsely populated regions, suggesting that the magnitude of such overestimation might be attenuated in this specific case. Future investigations should prioritize the integration of high-resolution land use/land cover change data (e.g., FROM-GLC, ESA CCI-LC), the Human Footprint Index, and forest structural parameters to enhance model accuracy and ecological realism [60,61].

## 5. Conclusion

This study identified the precipitation during the driest month (bio_14) as the primary driver of *K. longipedunculata*’s distribution, with topographic elevation (200–2000 m) playing a key role in forming a water-topography buffering mechanism that ensures the long-term stability of its core mountainous habitat in central Jiangxi (Yongfeng-Anfu-Jishu). Under future climate changes, low suitability habitats would sharply decline under SSP126-2050s and SSP585-2090s scenarios, elevating risks for peripheral habitats, whereas medium suitability areas exhibit widespread expansion, emerging as critical zones for distributional reorganization. The minimal fluctuations (<±8%) in high suitability zones showed the underscoring of the resilience of montane core refugia. However, ongoing vigilance against northward and upward habitat shifts driven by global warming remains imperative. To mitigate these risks, adaptive strategies should prioritize the conservation of core habitats in Jiangxi and the development of drought-tolerant germplasm sourced from northern marginal populations. The newly identified suitable habitats in western Hunan and northern Guangxi present viable opportunities for assisted migration or innovative cultivation initiatives. This study established mechanistic thresholds for pivotal climatic drivers, creating an evidence base for sustainable resource governance. Future models integrating anthropogenic variables, such as land-use change will enhance the predictive accuracy of habitat suitability frameworks, ultimately optimizing conservation prioritization and cultivation planning.

## Supporting information

S1 Data Kadsura longipedunculata. (CSV)
